# Moving to Music: Effects of Heard and Imagined Musical Cues on Movement-Related Brain Activity

**DOI:** 10.3389/fnhum.2014.00774

**Published:** 2014-09-26

**Authors:** Rebecca S. Schaefer, Alexa M. Morcom, Neil Roberts, Katie Overy

**Affiliations:** ^1^SAGE Center for the Study of the Mind, University of California, Santa Barbara, CA, USA; ^2^School of Philosophy, Psychology and Language Sciences, University of Edinburgh, Edinburgh, UK; ^3^Clinical Research Imaging Centre (CRIC), Queen’s Medical Research Institute, University of Edinburgh, Edinburgh, UK; ^4^Institute for Music in Human and Social Development, Reid School of Music, Edinburgh College of Art, University of Edinburgh, Edinburgh, UK; ^5^Don Wright Faculty of Music, Department of Music Education, University of Western Ontario, London, ON, Canada

**Keywords:** fMRI, music, music imagery, cued movement, neurorehabilitation

## Abstract

Music is commonly used to facilitate or support movement, and increasingly used in movement rehabilitation. Additionally, there is some evidence to suggest that music imagery, which is reported to lead to brain signatures similar to music perception, may also assist movement. However, it is not yet known whether either imagined or musical cueing changes the way in which the motor system of the human brain is activated during simple movements. Here, functional magnetic resonance imaging was used to compare neural activity during wrist flexions performed to either heard or imagined music with self-pacing of the same movement without any cueing. Focusing specifically on the motor network of the brain, analyses were performed within a mask of BA4, BA6, the basal ganglia (putamen, caudate, and pallidum), the motor nuclei of the thalamus, and the whole cerebellum. Results revealed that moving to music compared with self-paced movement resulted in significantly increased activation in left cerebellum VI. Moving to imagined music led to significantly more activation in pre-supplementary motor area (pre-SMA) and right globus pallidus, relative to self-paced movement. When the music and imagery cueing conditions were contrasted directly, movements in the music condition showed significantly more activity in left hemisphere cerebellum VII and right hemisphere and vermis of cerebellum IX, while the imagery condition revealed more significant activity in pre-SMA. These results suggest that cueing movement with actual or imagined music impacts upon engagement of motor network regions during the movement, and suggest that heard and imagined cues can modulate movement in subtly different ways. These results may have implications for the applicability of auditory cueing in movement rehabilitation for different patient populations.

## Introduction

The connection between musical rhythm and movement is an intuitive one, of which the most obvious manifestation is the widespread inclination to move to music. Coordinated movement necessarily depends on precise timing mechanisms and the inherent temporal structure of music can be used to make movements more regular (cf. Thaut et al., 2002; Bood et al., 2013). The connection between rhythm perception and movement has been demonstrated in cognitive neuroimaging studies: motor areas such as premotor cortex (PMC), basal ganglia, and cerebellum have been found to be activated when people listen to musical rhythms (Grahn and Brett, 2007; Chen et al., 2008). Motor network activation has also been found when people imagine music; additional to superior temporal and inferior frontal activation, the pre-supplementary motor area (pre-SMA), and PMC (both in BA6) have consistently been implicated in music imagery, and some studies have reported striatal and cerebellar activation (cf. Halpern and Zatorre, 1999; Leaver et al., 2009; Herholz et al., 2012). The neural activations of music imagination and music perception have also been found to show commonalities, both in terms of regional specificity measured using positron emission tomography (PET) or functional magnetic resonance imaging (fMRI) (cf. Halpern and Zatorre, 1999; Kraemer et al., 2005; Herholz et al., 2012) and in terms of temporal activation patterns measured using magneto-encephalography (MEG) or electro-encephalography (EEG) (Herholz et al., 2008; Schaefer et al., 2009, 2011a,b). Notably, the degree of such shared activation appears to vary with the complexity of the imagined musical stimulus, with more shared activation for simple stimuli than for complex stimuli such as ecologically valid music (Schaefer et al., 2013). The overlap between imagery and perception is not unique to the auditory modality; both visual and movement imagination have been found to activate modality-specific brain regions (Kosslyn et al., 2001; Pfurtscheller et al., 2006), and imagery content can even be decoded from the brain responses in visual and motor areas (Cichy et al., 2012; Oosterhof et al., 2012). The implication is that aspects of the imagined stimulus or action are being processed much in the way the actually perceived stimulus or performed action might be, usually with a weaker signature in modality-specific regions (Kosslyn et al., 2001) and together with additional, modality non-specific frontal and parietal activations that are related to imagery quality or vividness, likely involving memory and attentional processes related to the cognitive effort of imagining (Daselaar et al., 2010). Differences in activation patterns related to auditory imagery have also been reported between people with high- and low-imagery ability, showing differences in auditory and frontal regions (Herholz et al., 2012) although this vividness effect was not found in a previous study on non-musical auditory imagery (Olivetti Belardinelli et al., 2009).

Movement cueing refers to the use of an auditory stimulus to guide the temporal structure of a movement. The use of music as a cue for movement is common in recreational activities such as dancing or coordinated actions such as marching, and has been described as increasing athletic endurance (Karageorghis et al., 2010) and decreasing perceived exertion (Bood et al., 2013). However, aligning movement to a complex auditory stimulus such as music also involves cognitive operations such as periodicity detection and prediction (or beat induction, Honing, 2012) and tempo tracking. Auditory cueing is also used in rehabilitation of a range of movement disorders (Schaefer, in press), and several studies have indicated positive effects of music in movement rehabilitation. A meta-analysis of music-based gait interventions for Parkinson’s disease (PD) showed small but significant effects on specific gait-related outcome measures (de Dreu et al., 2012), and significant effects have also been reported for rehabilitation of gait after stroke (Bradt et al., 2010). However, a worsening of gait has been reported in patients with Alzheimer’s disease (AD) when walking to auditory cues (Wittwer et al., 2013), interpreted as related to impaired executive functioning, implying that attentional resources are necessary when aligning movements to music.

A small number of reports examine the use of imagined music as a cue for movement in rehabilitation settings. Schauer and Mauritz (2003) reported anecdotally that their music-based intervention was causing people to imagine the learned music while they walked, and more recently, imagined singing was successfully used to regularize gait in a small group of PD patients (Satoh and Kuzuhara, 2008). These reports suggest that a musical cue can potentially be endogenously generated through music imagery; however, there is currently no direct neural evidence to suggest that imagery might support movement in a similar way to perceived music.

When considering the possible mechanisms of movement cueing within the motor network of the brain, one possibility is that the motor network activations previously reported for musical rhythm perception (Grahn and Brett, 2007; Chen et al., 2008) may additively combine with the brain activation related to actual movements, leading to increased activation in basal ganglia, PMC, and (pre-)SMA. However, previous studies have found different rather than greater motor network activity when movement is cued by music compared to when it is carried out with no external cue, i.e., self-paced. Brown et al. (2006) used PET to directly compare music-cued dance-step foot movements in the supine position with the same movements in silence. This is to our knowledge the only brain imaging study, to date, to investigate movement cueing using naturalistic music stimuli, rather than a metronome, and compare it directly to self-paced movement. Results showed additional activation in lobule III of the cerebellar vermis during entrained relative to self-paced movement. This supports the longstanding notion that the cerebellum is involved in the timing of movement as well as in rhythm perception (Ivry and Keele, 1989; Schubotz et al., 2000). In a more recent fMRI study that directly compared cued and uncued conditions, using stepping movements and metronome stimuli, Toyomura et al. (2012) found greater left putamen activity for uncued movement relative to cued movement, which was attributed to self-pacing of the movement. The difference from Brown et al. (2006) findings may be due to the more recent study using either simpler movements (alternating foot raises as opposed to spatially organized dance steps), or may be due to simpler cueing stimuli (a metronome as opposed to tango music), or some interaction of these factors. Schaal et al. (2004), in a study focusing on rhythmic and discrete movements, reported that wrist flexions performed periodically to a metronome cue elicited similar, but weaker activation patterns in PMC to those elicited by self-paced movements. This suggests that the cue somehow facilitated the movement, leading to a reduced demand on the same neural resources. However, Schaal et al.’s study did neither focus on nor directly statistically compare, the cued and uncued movements, limiting possible interpretations. Collectively, brain imaging work has thus not yet provided clear indications of a neural basis for the potential clinical benefits of moving to music or imagined music.

In the current study, we used fMRI to evaluate the effects of musical cueing of a very simple movement on motor network activations, using an adaptation of the movement used by Schaal et al. (2004). We used two different cueing conditions: moving to music and moving to imagined music, with a control condition involving the same movement carried out without a cue, referred to as self-paced. We aimed to explore the activation of motor regions related to entraining movement to music, and the possible equivalence of using imagined and perceived music as cues. Possible differences between these conditions in terms of movement output were evaluated separately outside of the scanner in a behavioral study in a different group of participants. Based on the previous research findings reviewed above, a number of neural motor regions of interest are identified, namely, PMC (pre-)SMA, basal ganglia, and cerebellum. Considering the simple wrist flexion movement used here, reduced PMC activations were predicted to be found for cued (musical or imagined musical) compared to uncued movement (cf. Schaal et al., 2004). Based on previous results of musical cueing, cued movement was predicted to lead to activations in anterior cerebellum III (cf. Brown et al., 2006), and following the results of Toyomura et al. (2012), we expected that basal ganglia may be implicated in self-pacing movement. For music imagery without movement, the motor areas pre-SMA, PMC, and the cerebellum have been reported to be active (cf. Halpern and Zatorre, 1999; Leaver et al., 2009; Herholz et al., 2012). As the above-mentioned findings of reduced activation for imagery relative to perception only concerned modality-specific areas rather than activations found in the motor network, we hypothesized that, based on the reported shared activations between music perception and imagery, the activations in the pre-SMA, PMC, and the cerebellum when moving to music would be similar to those when moving to imagined music, contrasted with self-paced movement. Notably, Daselaar et al. (2010) reported bilateral striatum to be more active for (non-musical) auditory imagery than for perception, indicating that activation in this region may be increased for imagery-based cueing. The assessment of the movements with motion capture was predicted to show that for a simple movement such as wrist flexions, there would be no differences between conditions in terms of global movement parameters such as speed or range of movement.

## Materials and Methods

The fMRI and behavioral experiments were carried out in accordance with the code of ethical principles for medical research involving human subjects of the World Medical Association (Declaration of Helsinki), and was approved by the ethical committee of the University of Edinburgh and the West of Scotland Research Ethics Committee, UK, REC reference number 12/WS/0229.

### Participants

Seventeen volunteers (8 female, mean age 27.3 years, range 20–46), recruited through University of Edinburgh networks, took part in the fMRI experiment after giving informed consent. All participants were self-reported right-handed [mean shortened Edinburgh handedness inventory (EHI; for details see Stimuli and Materials)] score 74.6, SD = 19.8, all classifying as right-handed (>40) except for one who scored ambidextrous at 25 points, but identified as right-handed. All had <5 years of formal music training (mean 2.2, SD = 2.2) but listened to music at least three times a week and reported being able to imagine music, and had no known neurological impairments. For the behavioral experiment, 10 additional right-handed volunteers were recruited with the same recruitment criteria, of which 1 had to be excluded due to data loss and 1 due to having more musical training than reported at the time of recruitment, resulting in 8 behavioral participants (5 female, mean age 23.6, range = 20–45), mean shortened EHI 89.6 (SD = 12.0, all classifying as right-handed), and a mean of 1.1 years (SD = 1.5) of formal music training.

### Stimuli and materials

Two experimental conditions and one control condition were presented in blocks: flexing the wrists either to music (music condition), or while imagining music (imagined music condition), or without a cue (self-paced condition), respectively. The tempo was indicated through a visual count-in cue, which was identical for all conditions. The wrist flexions were bilateral and identical for both hands, with one dorsiflexion and one plantar flexion per second, starting the movement block with a dorsiflexion each time after the count-in. The music fragment that was used consisted of bar 9–16 (15.8–31.6 s) of Stevie Wonder’s “Another Star” (from “Songs in the Key of Life,” 1976, Motown Records), available as Supplementary Material. This fragment was chosen for its tempo [120 bpm, which is in the range of preferred tempi for repeated movements, see Moelants (2003)], the fact that it is a natural performance with some rhythmic complexity that it has a vocal melody but no words, thus not including semantic content, and is sung by both men and women, which we hypothesized to facilitate the imagery for the music fragment.

To assess the recruitment criteria in terms of general auditory imagery ability and hand preference, the auditory part of the shortened Betts’ questionnaire upon mental imagery (BQMI, Sheehan, 1967) and the EHI (Oldfield, 1971) were used. An exit questionnaire asked participants to rate the clarity and ease of the task on a five-point scale, give details of any prior musical training experience, and indicate when and how often they generally imagine music.

### fMRI experiment

#### Procedure

**Figure 1:**
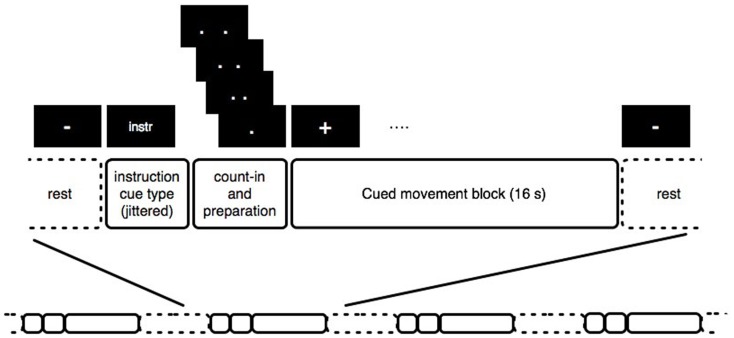
**Graphical representation of the stimulus structure**.

The procedure for the experiment is illustrated in Figure 1. Each block comprised a single sequence of wrist flexion movements, instructed to be performed with a maximal range within the limits of comfort, preceded by a cue indicating the movement condition. For each condition, the instructions were first presented for 1.5–2.5 s, randomly jittered so that the timing of the instruction was uncorrelated with the block onset. Then, the word “Ready?” appeared for 1 s, then a black screen for 1 s, followed by a visual count-in of four pairs of dots closing in to the center, presented at intervals of 500 ms, after which a “+” appeared on the screen for 16 s while the movements were performed. The change from a “+” to a “−” indicated that the block was finished, and the participant should rest until the next block started. The inter-block rest period lasted 13–14 s, and each participant performed 10 blocks of each condition in a randomized order. Prior to task performance, participants practiced the movement, then listened to the music fragment until they indicated that they were able to imagine it easily (with no one taking more than 10 min of listening), and finally practiced five trials with the experiment instruction screens presented on a laptop. In case of involuntary imagery of the music during the self-paced condition, participants were suggested the back-up strategy of imagining the sound of a metronome, however, none required this strategy during scanning – all reported having no involuntary music imagery during the self-paced condition. Once the participant had confirmed that they understood the experiment by performing each condition appropriately, they were positioned comfortably in the scanner. The stimuli were presented using Presentation^®^ software^1^ (Version 0.70). Visual stimuli were presented using Nordic NeuroLabs goggles, and standard Siemens headphones were used to present the music. The full session included a structural scan lasting 6 min followed by two 20-min functional scanning runs, of which this study was the second. The first is reported elsewhere, and involved the same movement, but cued with a metronome. In terms of possible sequential effects, this means the participants had some practice in performing the wrist flexions in the scanner, which, given the simplicity of the movement, would not be expected to result in differences in motor network activations, and in any case would be expected to affect each condition of the currently discussed experiment equally. After the scanning runs, participants filled in the BMQI, EHI, and exit questionnaire.

#### Imaging data acquisition and analysis

Anatomical and functional images were acquired at the Clinical Research Imaging Centre (CRIC) at the Queen’s Medical Research Institute (QMRI) of the University of Edinburgh, UK, using a 3-T Siemens Magnetom Verio scanner with 12-channel matrix headcoil. For the anatomical images, an MPRAGE sequence was used (160 slices, TR = 2300 ms, TE = 2.98 ms, FOV = 240 mm × 256 mm × 160 mm, voxel size = 1 mm^3^, TI = 900 ms, flip angle = 9°). Functional activations were assessed by acquiring T2*-weighted gradient-echo echo planar (EPI) images with blood oxygen level-dependent contrast (26 slices, TR = 1560 ms, TE = 26 ms, FOV = 192 mm × 192 mm × 130 mm, voxel size = 3 mm × 3 mm × 5 mm, flip angle = 90°, no gaps between the slices). The first six volumes were discarded to allow for T1 saturation effects.

#### Preprocessing

Data analyses were performed using Statistical Parametric Mapping software^2^ (SPM8, version 4191) with MATLAB 7.10 (The MathWorks, Natick, MA, USA). The preprocessing steps were as follows: first, an outlier detection procedure was applied to ensure data quality; outlier slices with values >7 SD above the mean were identified and scans were replaced with the mean of the 2 neighboring scans to remove any faulty scans (replacing an average of 0.8 volumes, maximum 3 per participant, out of a total of 604 volumes). After realigning the functional data to one of the middle scans of the run with B-spline interpolation, and saving a mean image and the motion parameters, the structural image was coregistered to the mean functional image. Spatial normalization to MNI space then followed the optimized VBM5 procedure (Ashburner and Friston, 2005) with the “New Segment” option in SPM8: the structural image was segmented into six tissue types, and the resulting forward deformation field then applied to the functional images, reslicing to 3 mm × 3 mm × 3mm voxels. Finally, the functional data were spatially smoothed using a 6 mm × 6 mm × 6mm full-width at half maximum (FWHM) Gaussian kernel.

#### Statistical analysis

Statistical inference employed a two-stage summary-statistic approach (Holmes and Friston, 1998; Penny and Holmes, 2006). First level general linear models (GLMs) for each subject were constructed with separate covariates for each of the three conditions (music, imagined music, and self-paced), modeled as sequences of 16 s blocks beginning at the onset times of each sequence of movement. Three additional covariates were included to account for the initial preparation and counting-in phase of each condition, modeled as sequences of 2 s blocks corresponding to the duration of the preparation time. This ensured that condition-specific movement effects of interest were not confounded with condition-specific movement preparation effects. Covariates were formed by convolution of block sequences with a canonical hemodynamic response function (HRF; Friston et al., 1998). Parameter estimates were then computed using the weighted least squares fit of the model to the data following pre-whitening with an AR(1) plus white noise model (Friston et al., 2002). Data for each session were high-pass filtered to 1/128 Hz and scaled to a grand mean of 100 across all voxels and scans within a session. Each GLM also included a session constant, and six movement regressors as covariates of no interest.

Second level analyses were conducted on first level contrast images, treating participants as a random effect. For each participant, three simple pairwise contrasts entered group-level one-sample *t*-tests, which compared parameter estimates between each movement condition (music and self-paced, imagined music and self-paced, music and imagined music). To focus on motor network effects, group analyses were conducted within a region of interest (ROI) mask created using the WFU Pickatlas toolbox (Maldjian et al., 2003). This mask comprised bilateral areas BA4, BA6, caudate, putamen, globus pallidus, motor thalamus (ventral anterior nucleus and ventrolateral nucleus; Mai and Forutan, 2012), and the entire cerebellum. Cluster significance was tested within the ROI mask using the AlphaSim tool included in the AFNI toolbox^3^ (Cox, 1996). This simulation indicated that with a cluster-forming threshold of 0.005, a family wise error (FWE) cluster correction at *P* < 0.05, required a minimum cluster size of 19 voxels. The locations of the clusters were determined using the probabilistic maps integrated in the SPM Anatomy Toolbox v1.8 (Eickhoff et al., 2005). The results of the ambidextrous participant were inspected in relation to the significant clusters, and were found not to show different results than the formally right-handed participants (as determined by the EHI).

### Behavioral experiment

#### Additional procedure and analysis

Except where noted, procedures for the behavioral experiment followed those described for the fMRI experiment, but the rest period between trials was now shortened to 5 s since a longer period of rest was not needed for behavioral data acquisition (see Figure 1). Participants assumed a supine position to mimic the fMRI set-up, with support pillows so that they could see a computer screen set-up in front of them and rest their arms while making the wrist flexion movements. An Ascension miniBIRD magnetic motion tracking system was used to capture the timing and extent of the wrist flexion, with a single 8 mm sensor attached to the middle finger of their right hand with medical tape (as a proxy for the movement of both hands) measuring with a sampling frequency of 100 samples per second. After 5 practice trials (during which the measurement was tested), 11 trials per condition were presented in a randomized order. This experiment included an additional condition in which movements were performed to a metronome, the results of which are discussed elsewhere. The experiment took approximately 20 min. Visual stimuli were presented using a 20″ computer screen, which was set-up in front of the participants who were in half supine position, and the sound was presented through stereo speakers (Genelec 1029A, 40 W, free field frequency response of 70 Hz – 18 kHz ± 2.5 dB) positioned to the sides of the screen and set at a comfortable listening level.

The data were analyzed in MATLAB 7.10 (The MathWorks, Natick, MA, USA). Using only the *z*-axis of the recording, movement blocks with obvious measurement artifacts (such as large spikes) were first removed by hand, leading to rejection of 5 blocks, leaving an average of 10.8 16-s movement blocks per condition (minimum = 8). The movement features analyzed were based on Schaal et al. (2004), and included the number of movements, range of the movements, mean period of a full flexion, and the mean and maximum velocity. Signals were first normalized to *z*-scores over all conditions, after which the number of movements, the (normalized) range, full back, and forth flexion period duration and velocity (displacement over time, mean, and maximum) were computed by extracting the dorsiflexions and plantar flexions for all individual trials and then averaging these over each condition and participant before averaging over the group. As there was unequal variance in all measures between conditions, the output measures are tested using the Friedman test (Friedman, 1937) as implemented in MATLAB, with *post hoc* comparisons tested separately.

## Results

### Questionnaire results

The results of the EHI and musical background questions are reported above in Section “Participants.” All participants (fMRI and behavioral) indicated that they were not highly familiar with the music fragment and found the task easy, while most participants indicated that they found the experiment enjoyable (14/17 for the fMRI group and 7/8 for the behavioral group). On the BQMI measure of imagery ability, which uses a 5-point scale (where 1 is high-imagery ability), the fMRI group had an average score of 2.05 (SD = 0.8), and the behavioral group had an average score of 2.48 (SD = 1.17). These are not statistically different between groups using the Mann–Whitney test, and both are slightly below the normative average of 3.01 (SD = 1.53), reported originally by Sheehan (1967), indicating somewhat better imagery vividness in these participants than average, in accordance with the recruitment criteria.

### Behavioral results

The mean number of movements, the mean amplitude of the wrist flexions, the mean period, and the mean and maximum velocity of the movements are shown for each condition in Table 1. Given that the tempo of the music (which was the same as the instructed tempo for the silent and imagery-cued conditions) was 120 bpm, the 16 s blocks should lead to 16 flexions and a mean period of 1 s. As the amplitudes (or extent of the movement) were normalized between participants they are reported in terms of the *z*-score, and both velocity measurements are thus in arbitrary units. The movements were found to be highly similar; the Friedman test revealed no statistically significant differences between the conditions for any of the outcome measures. These results indicate that large-scale differences in velocity or range were not found between the different movement conditions.

**Table 1:** **Behavioral results: wrist flexions for each cueing condition**.

Condition	Wrist flexions	Amplitude (*z*-scores)	Period (s)	Velocity (arbitrary units)	Maximum velocity (arbitrary units)
Self-paced	15.56 (1.37)	2.84 (0.07)	1.07 (0.09)	0.05 (0.005)	0.16 (0.005)
Music	16.09 (0.94)	2.83 (0.07)	0.99 (0.04)	0.05 (0.003)	0.18 (0.003)
Imagined music	16.12 (1.29)	2.85 (0.08)	1.02 (0.07)	0.05 (0.005)	0.17 (0.005)

*The number of movements, the mean amplitude, and mean period of each back and forth flexion, and mean and maximum velocity over the whole block of movement are shown with SD in brackets. Between-condition comparisons did not reveal any significant differences (*P* > 0.05, Friedman test)*.

### fMRI results

**Figure 2:**
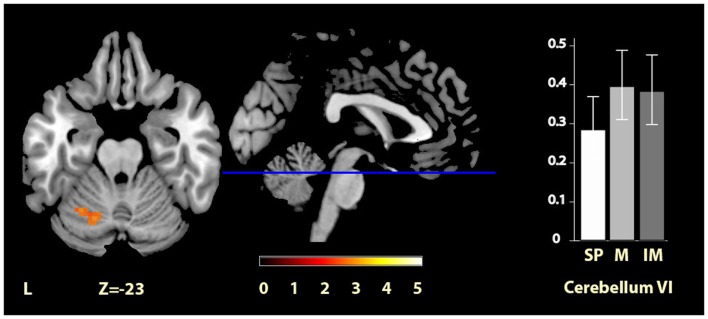
**Musically cued versus self-paced movement**. The section shows the left cerebellum VI region activation cluster for music relative to self-paced conditions, displayed on the MRIcron reference T1 image in MNI space, FWE cluster corrected at *P* < 0.05. The parameter estimate plot shows mean percent signal change of the self-paced (white, SP), music (mid-gray, M), and imagined music (dark gray, IM) conditions relative to rest in cluster peak voxel *x* = −27, *y* = −58, *z* = −23.

**Figure 3:**
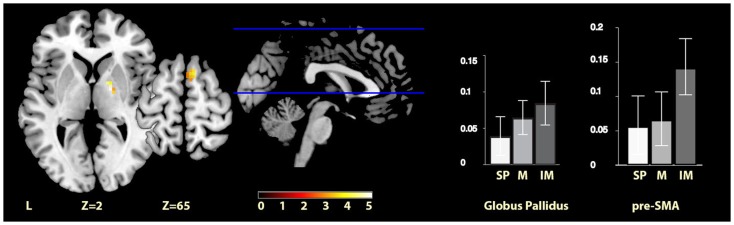
**Imagery-cued versus self-paced movement**. The sections show activation clusters in the right globus pallidus and pre-SMA for imagined music relative to self-paced conditions, displayed on the MRIcron reference T1 image in MNI space, FWE cluster corrected at *P* < 0.05. The parameter estimate plots show percent signal change of self-paced (white, SP), music (mid-gray, M), and imagined music (dark gray, IM) conditions relative to rest in cluster peak voxels *x* = 18, *y* = −4, *z* = 1 (globus pallidus) and *x* = 9, *y* = 11, *z* = 64 (pre-SMA).

**Figure 4:**
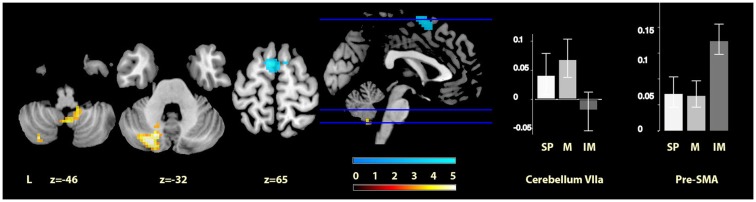
**Musically cued versus imagery-cued movement**. The sections show the cerebellar activations for music relative to imagined music conditions in orange and the pre-SMA activations of imagined music relative to music conditions in blue, displayed on the MRIcron reference T1 image in MNI space, FWE cluster corrected at *P* < 0.05. The parameter estimate plots show percentage signal change of self-paced (white, SP), music (mid-gray, M), and imagined music (dark gray, IM) conditions relative to rest in cluster peak voxels *x* = −24, *y* = −76, *z* = −32 (cerebellum VIIa) and *x* = −6, *y* = 1, *z* = 69 (pre-SMA).

Significant clusters of activity within the regions of interest are summarized in Table 2 and illustrated in Figures 2–4. There was significantly increased activity for the music condition relative to the self-paced condition in a cluster in the left cerebellum, lobule VI (Figure 2), but no region showed significantly increased activity for the self-paced condition relative to the music condition.

**Table 2:** **Regions showing significant between-condition differences in BOLD activation**.

Contrast	Anatomical region	Cluster size	Peak voxel (*x*, *y*, *z*)	*z*-Value
Music > self-paced	Left cerebellum VI	28	−27, −58, −23	2.97
			−18, −64, −23	2.95
Imagined music > self-paced	Right globus pallidus	19	18, −4, 1	5.24
	Right pre-SMA	20	9, 11, 64	3.17
			12, 5, 73	3.40
Music > imagined music	Left cerebellum VIIa	218	−24, −76, −32	4.03
			−18, −70, −32	3.98
			−30, −76, −38	3.97
	Cerebellar vermis IX	37	3, −55, −47	3.28
			12, −43, −47	3.11
Imagined music > music	Bilateral Pre-SMA	117	−9, 8, 67	4.16
			12, 8, 67	3.20

*See Section “Statistical Analysis” for details of analysis and thresholding*.

The comparisons between the imagined music condition and the self-paced condition revealed significant activity increases for imagined music relative to self-paced in the right pre-SMA and in the right globus pallidus (see Figure 3). Again, no region showed significantly increased activity for self-paced relative to imagined music.

Finally, comparisons between the two cued conditions (music and imagined music), showed music-specific activations in left cerebellum lobule VIIa and the vermis of lobule IX, whereas for imagery, activation was seen in bilateral pre-SMA (see Figure 4). The cerebellar cluster did not overlap with the cerebellar activation identified in the contrast between music and self-paced, but the pre-SMA cluster did show five voxels overlap with that found in the contrast between imagined music and self-paced.

## Discussion

In the current study, brain activity within motor regions during a simple wrist flexion movement task was found to vary according to how this movement was cued. We observed differential responses in cerebellum, globus pallidus, and pre-SMA according to whether wrist flexion movements were performed to music, to imagined music or were self-paced. Self-paced movement did not elicit significant additional activity relative to either of the cued conditions. A closely matched behavioral study showed that these neural differences were not related to gross differences in performance: the number, amplitude, and velocity of the movements showed no significant differences between conditions. Thus, in the cued conditions, increased activations in specific motor areas were associated with a similar behavioral output, suggesting differing neural processes to lead to the same movement.

Our data provide a direct comparison of musically cued and uncued movements while keeping movement complexity minimal, and both converge and diverge from previous findings. As predicted, we observed pre-SMA activation during movement cued by musical imagery relative to heard music as well as relative to self-paced movements. However, the hypothesized activity increase in anterior cerebellum III and activity reduction in PMC for musically cued compared with self-paced movement were not seen. The areas that were active in the cued conditions over the self-paced condition (left cerebellum VI for music, and right pallidum and right pre-SMA for music imagery) are all regions that have previously been implicated in finger tapping tasks cued by auditory pacing signals (Witt et al., 2008), but have not previously been found in direct comparisons of cued to uncued movement. We now consider these findings in turn.

### Musical cueing

Performing wrist flexions to music, as compared to self-cued movement, yield activation in lobule VI of the left cerebellum. This area has previously emerged as involved in musical processing (cf. Peretz and Zatorre, 2005; Alluri et al., 2012), but is also involved in a variety of other cognitive and motor tasks (Stoodley and Schmahmann, 2009). Our finding differs from those of Brown et al. (2006) PET study, which examined activity for patterned dance steps performed to natural music contrasted to the same steps but self-paced, and reported cerebellar activation in lobule III of the vermis, which was interpreted as specific to entrainment to musical rhythm. Although this study is to our knowledge the only other study directly comparing musically cued movement to uncued movement, there are several basic differences in the study design that could cause divergence from these previous findings. It is possible that the very simple movement used in the current experiment, based on a previous research report (Schaal et al., 2004), may lead to different cueing effects from more complex movements. Recurring wrist flexions are easy to perform without too extensive effort or attention, fitting with the rationale for the current experiment, which had the aim of assessing the effect of auditory cues for simple movements, whereas it may be the case that for more complex movements, such as the tango dance steps used in Brown et al. (2006), the effects of auditory cues are different, involving the cerebellar vermis, or alternately that the musical style is relevant for the effect of a cue on motor network activation. The increased sample size used in the current study (*N* = 17) as compared to previous work on cued movement [studies by Brown et al. (2006), Schaal et al. (2004), and Toyomura et al. (2012) had Ns of 10, 11, and 12, respectively] lends some support to the current findings as a robust reflection of mechanisms of simple movements cued by a musical stimulus.

In previous studies, multiple motor areas including cerebellum VI have been reported as being activated during rhythm perception [such as cerebellum VIII (pre-)SMA, PMC, putamen, see Chen et al. (2008), Grahn and Brett (2007)]. Thus, one possibility is that the cerebellum VI activation found in the current study is purely due to music listening. In that case, it is of interest that this cerebellar area was the only motor region found to be significantly activated by music perception in the current context of continuous movement, suggesting that, whereas the perception-related activation in other motor regions does not persist while performing a movement, this is not the case for the cerebellum VI region. Alternatively, this region may respond specifically to musically cued movement, and be crucial for aligning movement to sound. Based on the data presented here, it is not possible to distinguish between these potential explanations. However, it appears likely that cerebellum VI, with its connections to PMd and M1, as well as frontal and parietal areas (Bernard et al., 2012), acts as a hub of connectivity linking perceptual and motor processes [much like the anterior cerebellar activation was interpreted in Brown et al. (2006)], and has a distinct role compared to other movement-related regions involved in music processing. Future studies may reveal further motor region effects for cued movements of varying complexity, as here we only examined the simplest case, a regularly recurring, easy movement.

### Imagery-based cueing

When comparing movement cued by imagery to self-paced movement, we found significant clusters of activity in two regions: one in the right pre-SMA and one in the right globus pallidus. The former was expected from previous literature on music imagination (cf. Halpern and Zatorre, 1999; Leaver et al., 2009; Herholz et al., 2012) and is found here to persist in the context of movement. In previous literature, this activation has been proposed to relate to sequential control through chunking and memory processes (Leaver et al., 2009), and has also been implicated in free musical improvisation of melodies and rhythms (de Manzano and Ullén, 2012), which in a different way also includes imagery, memory, and sequencing processes. More generally, pre-SMA is associated with self-initiation of actions and cognitive involvement in action (Nachev et al., 2008). Although not reported consistently, activation in the pallidum has also been noted previously in the context of music imagery, specifically vividness in anticipatory imagery (Leaver et al., 2009), while striatal activation has been reported for vividness of (non-musical) auditory imagery as compared to perception (Daselaar et al., 2010). The current finding of pallidum activation is specifically interesting in the context of movement cueing in clinical situations, as the globus pallidus is one of the main sites for which deep brain stimulation (DBS) has been found to be effective in reducing motor impairments [another site being the subthalamic nucleus, cf. Follett et al., 2010]. In PD, this stimulation has for example been reported not only to reduce tremor and improve gait (Follett et al., 2010) but also to reduce symptoms in various types of dystonia (Kupsch et al., 2006; Walsh et al., 2013), Huntington’s disease (Edwards et al., 2012), and Tourette’s syndrome (Priori et al., 2013). The finding that this area is especially active during imagery-cued movement needs further investigation and replication before a robust connection with these patient populations can be established; however, this first brain imaging result of moving to imagined music shows promise for further development of paradigms using music imagery in clinical situations. The beta and gamma frequency bands of electrical activity in the internal globus pallidus (as measured in cervical dystonia patients with implanted electrodes) are modulated by the preparation of self-initiated movements and the execution of both self-initiated and externally triggered movements (Tsang et al., 2012). For PD, bilateral thalamus and pallidum size are positively correlated with disease duration (Geevarghese et al., 2014), and altered pallidal–frontal processing is associated with executive dysfunction (Dirnberger et al., 2005). Moreover, patients with focal basal ganglia lesions are reported to be affected in the ability to execute a steady sequence of periodic actions (Schwartze et al., 2011). The finding that this area is more active for imagined music than during self-paced sustained movement, but not significantly different from musically cued movement may be related to the relative endogenous effort involved in pacing movement through imagery.

### Comparison of musical cueing and imagery-based cueing

Directly contrasting the two cued movement conditions showed significantly greater activity for musical cueing in two main clusters in the cerebellum (i.e., lobule VIIa of the left hemisphere and lobule IX of the vermis), and significantly greater activity for imagery-based cueing in bilateral pre-SMA. The latter result was predicted on the basis of previous literature on music imagination, and the robust difference with musical cueing indicates that the pre-SMA was uniquely significantly activated by imagined music. Regarding our hypothesis of equivalence between music and imagined music, when considered alongside the results discussed above (see Musical Cueing and Imagery-Based Cueing), the cerebellum VI and pallidum regions, which responded to musical and imagery-based cueing compared with self-paced movement, respectively, did not show reliably different activity when these two conditions were contrasted directly with one another. This is supported by inspection of the parameter estimates in both regions, suggesting intermediate responses for the contrasts with self-paced movement for imagined music for cerebellum VI and music for the pallidum. Thus, our data are consistent with the hypothesis that moving to music and to imagined music engages some of the same parts of the movement network, and also suggests that these regions are engaged to differing degrees.

In terms of the unpredicted activation differences that were found for music-based cueing as compared to imagery-based cueing; the cerebellar left lobule VII and vermis IX activations could be interpreted as relating to temporal prediction. A recent study by Pecenka et al. (2012) reported left cerebellum VIIa activation to correlate negatively with temporal prediction accuracy while timing movement to a metronome with a slightly varying tempo (although their other reported activations of SMA and precentral gyrus, which are also included in our search volume, were not found for our music condition). Additionally, cerebellum VIIa has been implicated in perceptual prediction without moving (O’Reilly et al., 2008). The activation in lobule IX of the vermis, reportedly connecting to superior temporal gyrus (Bernard et al., 2012), may also be related to perceptual processes during music listening. This finding offers some exploratory detail on the different role that cerebellar lobule VI may have as opposed to lobule VIIa and IX in aligning movement to auditory cues, in that cerebellum VI appears to be more involved in both external and internal rhythm processing, whereas lobule VIIa and IX may be specifically implicated in perceiving versus self-generating a rhythmic stimulus. Future studies will be necessary to verify and refine this interpretation.

### Limitations

A number of study limitations need to be kept in mind when considering these interpretations. Firstly, the current design did not include trial-to-trial vividness measures, and we thus relied on self-report from the participants after scanning that they were successfully imagining the music. This is a potential limitation as previous results indicate that imagery ability and vividness may affect the brain signatures of imagery (cf. Olivetti Belardinelli et al., 2009; Herholz et al., 2012). However, the regions previously reported to be implicated in imagery vividness were not motor areas and thus outside our regions of interest for the current study. Moreover, all participants in our study were pre-selected for imagery skills, all participants reported imagining the music easily, and we found significant differences in brain activity between imagery and self-paced conditions, suggesting that the manipulation was successful. A second potential limitation of the study is that, although the movements were evaluated behaviorally outside the scanner that sample size was relatively small and the measures of amplitude, period, velocity, and number of completed flexions were somewhat crude, possibly missing more subtle aspects of movement, such as movement fluidity or jerk. Such differences have been previously reported for more complex cued movements that have longer trajectories (e.g., Thaut et al., 2002). Nevertheless, there is no reason to assume that differences in these subtle aspects of the currently used movement, if present, would explain the differences in the brain activity we observed. Finally, since our results were obtained from simple movements carried out by healthy volunteers, further research will be needed to assess how the differences in neural processing are affected by either more complex movements or by the specific neural deficits of particular patient groups. Future neuroscientific and behavioral investigations aimed at detecting subtle differences in movement due to heard and imagined musical cueing will be of substantial converging interest for interpreting the present findings and those of the clinical studies.

### Conclusion and future directions

The current work presents, a first step in researching the neural mechanisms of moving to heard and imagined music, using ecologically valid music and simple movements. A cognitive task – music imagination – is shown to impact upon the activation of the motor network of the brain, in line with anecdotal clinical reports of imagined music facilitating movement, implicating brain areas in the cerebellum and basal ganglia long known to be important for the timing of movement. Thus, cognitive aspects of music processing, likely involving some representation of the musical rhythm, are demonstrated to affect movement processing at the level of the motor network of the brain.

Future work may benefit from investigating different styles of music, taking into account personal music preference as well as specific rhythmic characteristics of the musical stimulus. Additionally, other movements may be examined, using different limbs, different degrees of complexity, or the extent to which the movements are rhythmic or discrete in nature. This study only employed one musical extract, while recent behavioral work has shown that style of music can modulate the effects of cueing in terms of movement vigor (Leman et al., 2013), suggesting that this difference in vigor may also lead to more extensive motor network activation. Furthermore, the ability to move to an auditory cue appears to differ across individuals (Tierney and Kraus, 2013), for which test batteries of synchronization skills have recently been developed (Farrugia et al., 2012; Fujii and Schlaug, 2013). Thus, also taking individual differences into account for paradigms using both heard and imagined cueing may inform the usefulness of auditory cues in movement rehabilitation for specific patient groups, ideally permitting selection of cues or cueing strategies that are optimally useful given a specific neurological problem. The usability of music imagination as a cue will depend fully on the imagery ability of a specific patient group or individual, which can currently be assessed through imagery ability measures such as the BQMI questionnaire used here, similar to those used for movement imagery in movement rehabilitation [for an example with stroke patients, see Malouin et al. (2007)]. Systematic clinical evaluation of the applicability of moving to heard and imagined music is necessary to evaluate whether the current fMRI findings have consequences for the specific patient groups that could benefit from musical or imagined musical cueing.

## Conflict of Interest Statement

The authors declare that the research was conducted in the absence of any commercial or financial relationships that could be construed as a potential conflict of interest.

## Supplementary Material

The Supplementary Material for this article can be found online at http://www.frontiersin.org/Journal/10.3389/fnhum.2014.00774/abstract

## Footnotes

^1^http://www.neurobs.com

^2^www.fil.ion.ucl.ac.uk/spm/software/spm8/

^3^http://afni.nimh.nih.gov/afni/
